# Alignment of Key Stakeholders’ Priorities for Patient-Facing Tools in Digital Health: Mixed Methods Study

**DOI:** 10.2196/24890

**Published:** 2021-08-26

**Authors:** Courtney Rees Lyles, Julia Adler-Milstein, Crishyashi Thao, Sarah Lisker, Sarah Nouri, Urmimala Sarkar

**Affiliations:** 1 Division of General Internal Medicine Department of Medicine University of California San Francisco San Francisco, CA United States; 2 Center for Vulnerable Populations Department of Medicine University of California San Francisco San Francisco, CA United States; 3 Department of Epidemiology and Biostatistics University of California San Francisco San Francisco, CA United States; 4 Center for Clinical Informatics and Improvement Research Department of Medicine University of California San Francisco San Francisco, CA United States; 5 Division of Palliative Medicine Department of Medicine University of California San Francisco San Francisco, CA United States

**Keywords:** medical informatics, medical informatics apps, information technology, implementation science, mixed methods

## Abstract

**Background:**

There is widespread agreement on the promise of patient-facing digital health tools to transform health care. Yet, few tools are in widespread use or have documented clinical effectiveness.

**Objective:**

The aim of this study was to gain insight into the gap between the potential of patient-facing digital health tools and real-world uptake.

**Methods:**

We interviewed and surveyed experts (in total, n=24) across key digital health stakeholder groups—venture capitalists, digital health companies, payers, and health care system providers or leaders—guided by the Consolidated Framework for Implementation Research.

**Results:**

Our findings revealed that external policy, regulatory demands, internal organizational workflow, and integration needs often take priority over patient needs and patient preferences for digital health tools, which lowers patient acceptance rates. We discovered alignment, across all 4 stakeholder groups, in the desire to engage both patients and frontline health care providers in broader dissemination and evaluation of digital health tools. However, major areas of misalignment between stakeholder groups have stymied the progress of digital health tool uptake—venture capitalists and companies focused on external policy and regulatory demands, while payers and providers focused on internal organizational workflow and integration needs.

**Conclusions:**

Misalignment of the priorities of digital health companies and their funders with those of providers and payers requires direct attention to improve uptake of patient-facing digital health tools and platforms.

## Introduction

While the US private sector has invested billions in digital health companies [1], and there have been rapid technological advancements [2], the majority of patients do not use digital health tools [3]. Broad public opinion surveys find that while 79% of Americans have searched for health information online, less than one-quarter of respondents had ever used patient-facing digital health tools, such as mobile device tracking or wearables [4,5]. Furthermore, the lowest rates of patient uptake of digital health tools are among underserved populations (such as low-income individuals) and chronically ill seniors [6,7]—these are populations with the highest overall burden of disease who might have the most to benefit from digital tools [5,8,9]. Finally, the impact of patient-facing digital health tools on clinical outcomes is not strong. Most privately developed patient-facing digital health tools lack an evidence base, and those with findings reported in peer-reviewed literature have mixed evidence of success [10-12]. Even where evidence exists, it often does not mature or scale; for example, despite evidence supporting the efficacy of diabetes self-management apps (such as those to assist and support users in tracking blood sugar levels, diet, and other behaviors), there are few long-term effectiveness studies [13], and few platforms have been implemented at scale across health care systems or nationwide.

In the midst of low adoption rates and limited broad scale evidence of effectiveness, investment in digital health companies continues to expand [1]—with a record $5.4 billion invested in the first half of 2020 during the recent COVID-19 pandemic [14]—underscoring the strong interest in and anticipated potential of using digital health tools to drive transformation. There have been large recent venture capitalist investments [15] specific to patient-facing digital health investment, such as in patient chronic disease management. The current state of digital health thus reflects an apparent disconnect between large financial investments in and population-level clinical and health gains from such tools [16]. However, little research has been performed to characterize and understand the root causes of this disconnect [17].

A deeper implementation-based understanding—of the priorities of private sector stakeholders’ (who are driving much of the investment in digital health) and those of health care leaders (who are implementing digital health)—is critical to drive the broad use and impact of patient-facing digital health tools. Specifically, a better understanding of how stakeholders’ perceptions of digital health priorities, opportunities, and gaps converge or diverge could serve to align the interests of key stakeholders in fostering digital health approaches that work across diverse populations and improve public and population health [17]. Therefore, we sought to systematically investigate key stakeholder groups’ perceptions of adoption and effectiveness of patient-facing digital health tools.

## Methods

### Design

We pursued a 2-phase primary data collection process in which we conducted open-ended interviews and then conducted a focused structured survey among 4 key stakeholder groups in the digital health ecosystem. We collected qualitative and quantitative data on perceptions of the patient-facing digital health ecosystem. We defined patient-facing digital health tools as privately developed apps (and devices linked to apps, such as wearables) rather than electronic health record tools such as patient portals, which have followed a different implementation pathway, or broad educational websites such as MedlinePlus, which serve as a resource rather than an intervention platform. We sampled individuals in 4 stakeholder groups to reflect the investment or development perspective (venture capitalists and digital health companies) and the perspective of those purchasing and deploying the tools (payers, which included health plans and self-insured employers, and health care providers or leaders). While patients are another key audience in this ecosystem, there is a large body of evidence focused on patient interests in and barriers to using digital health tools [6,18-20]. We, therefore, add to the existing evidence base by focusing on the remaining stakeholders in this study. The University of California San Francisco Institutional Review Board approved this study (18-25418).

### Conceptual Framework

We used an implementation science theoretical framework to identify areas of alignment and misalignment across stakeholder groups. The Consolidated Framework for Implementation Research (CFIR) comprises a set of domains designed to guide understanding of which practices, programs, or tools work and why across different contexts [21,22]. CFIR domains include the outer setting (events happening outside implementation, such as regulation), the inner setting (specific characteristics of health care organizations driving patient-facing digital health rollout), intervention characteristics (functionality and usefulness of the digital health tools, implementation processes, and individuals (patient or provider skills, knowledge, and beliefs). Given the multifactorial nature of the digital health ecosystem, CFIR allowed us to summarize data within and across multiple interacting domains and processes that could factor into successful implementation of patient-facing digital health tools and collectively influence uptake, effectiveness, and sustained use of patient-facing digital health.

### Sample

We used expert networks in combination with snowball sampling to identify stakeholders for this study [23]. First, we identified experts and leaders within each stakeholder group and met regularly to brainstorm and compose the original participant outreach list. Then, we asked for additional contacts or recommendations from each interviewee. We sought to recruit 5 participants from each of the 4 stakeholder groups and for at least 2 out of the 5 participants to have expertise with Medicaid/Medicare or population health to ensure that issues pertaining to digital health equity were addressed. For example, we interviewed both safety net health plans and providers as well as those treating or focusing on privately insured populations and a mix of companies and venture capitalists with experience in chronic disease or population health-focused products.

### Key Informant Interviews

We invited interview participants by email, and then scheduled and conducted individual hour-long interviews (by phone or video conference). The interview process lasted from March to December 2019, with 4 authors leading interviews; an additional author was present for notetaking. Participants provided verbal consent. All interviews were recorded and professionally transcribed.

Our semistructured interview guide (Multimedia Appendix 1) covered current patient engagement with digital health tools, factors affecting the development and adoption of patient-facing digital health solutions, health care system’s climate for digital health implementation, and broad health policy and regulatory environment for digital health products. While the interview guide was structured broadly around the CFIR topics, we used open-ended topic exploration rather than specific framework domains or descriptions to drive the conversations. Furthermore, we specifically focused a portion of every interview on broad patient characteristics, such as age, socioeconomic status, and language, that have an impact on digital health tool use, given that there is literature on disparities among key patient groups [3,7,8].

### Qualitative Analysis

Three members of the team coded 19 transcripts using the qualitative analysis software (Dedoose, version 9.0.17; SocioCultural Research Consultants LLC). The team first read the transcripts to deductively formulate codes mapped to top-level CFIR domains. Each CFIR domain was then examined across the 4 stakeholder groups, with an approach informed by a descriptive qualitative approach [24], given that there was no existing literature that directly compared perspectives among these diverse groups. Then, 2 team members individually recoded 4 of the transcripts using the refined codebook and inductive coding as new ideas emerged—often as concepts within each CFIR domain area. We referred any disagreements about coding to the entire study team for discussion to ensure that we followed a group-based process for data interpretation. Our coding collapsed inner setting and implementation processes into a single domain, given that there was variation among the stakeholders in the on-the-ground implementation experience of digital health tools (eg, venture capitalists had experience with workflows but would not have had extensive experience with individual health care system processes to elaborate on these concepts in depth). We also expanded coding to separately identify themes within the characteristics of individuals domain to generate information about both patient and provider perspectives, given that the beliefs and capabilities of both end users were described robustly during interviews. After finalization of the codebook, we divided and independently coded the remaining 15 transcripts.

In the second phase of qualitative analysis, we used direct comparison of the codes and exemplar quotes across stakeholder groups in order to generate the overarching themes that cut across interview topics and CFIR domains. All team members were involved in this process, which also occurred iteratively as analysis unfolded.

### Survey

We planned a second, structured phase of data collection to enable triangulation of the qualitative analysis. More specifically, to assess of the importance of each CFIR domain in the development and adoption of patient-facing digital health solutions, we developed a short survey. We sent the surveys and collected survey responses by email. Surveys were sent to all participants whom we previously interviewed, as well as to 1 additional digital health company and 4 additional payer organizations (to replace 4 interviewees who were unable to continue participating).

**Figure 1:**
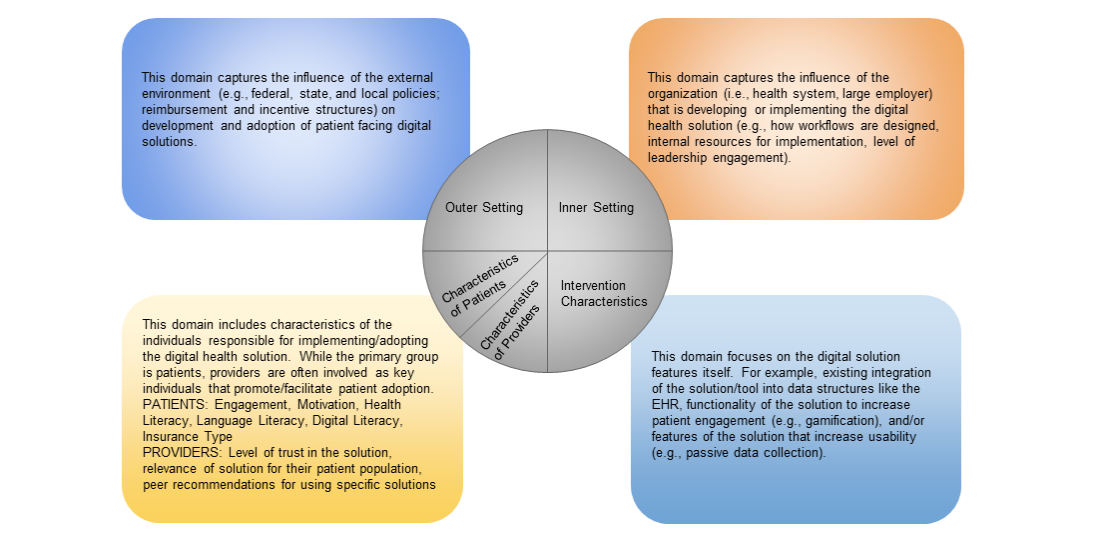
Domains that affect the development and adoption of patient-facing digital health solutions.

In the survey, we defined and explicitly named the CFIR domains that we used in the qualitative analysis: outer setting, inner setting, intervention characteristics, characteristics of the patient, and characteristics of the provider. Figure 1 displays the definitions shared with participants that explained CFIR domains and concrete examples of each domain from the qualitative analysis. We asked participants to allocate 100 points across CFIR domains on 2 survey items: (1) the extent to which the CFIR domain posed a challenge to the overall success and development of patient-facing digital health tools and (2) the extent to which the CFIR domain factored into the participant’s daily professional decisions.

### Survey Analysis

We calculated summary statistics (medians and interquartile ranges, given the small sample size and potential outlier responses) by CFIR domains. We compared stakeholder responses using radar charts and Mann–Whitney nonparametric tests.

## Results

### General

In total, we engaged 24 individuals across stakeholder groups, participants from 5 health-focused venture capitalists, 5 digital health companies, 8 payer organizations (including 3 focused on Medicaid and 4 commercial plans with a mix of private and Medicare/Medicaid products), and 6 providers (Table 1).

**Table 1:** Participant characteristics.

Stakeholder			Organization				
		Type		Region		Size (number of employees)	
**Venture capitalists**							
	Partner	Mature VC firm		West		51-100	
	Executive chairperson	Mature VC firm		West		11-50	
	Investor	Early-stage angel investor		West		≤10	
	Partner	Early-stage VC firm		West		11-50	
	Entrepreneur in residence	Early stage VC firm		West		≤10	
**Digital health companies**							
	Chief information officer	Series B company		West		11-50	
	Senior director of health plans	Series D company		West		251-500	
	Senior vice president^a^	Preseed company		West		≤10	
	Co-founder	Preseed company		Northeast		≤10	
	Senior vice president^b^	Seed company		West		11-50	
**Payer organizations**							
	Chief medical officer^a^	Medicaid health plan		West		251-500	
	Senior manager	Large employer organization		West		11-50	
	Chief medical officer^a^	Medicaid health plan		West		251-500	
	Senior vice president and chief digital officer^a^	Private insurance plan		Midwest		>10,000	
	Health informatics medical director^b^	Private insurance plan		West		11-50	
	Senior vice president^b^	Private insurance plan		West		5001-10,000	
	Chief executive officer^b^	Private insurance plan		Southeast		1001-5000	
	Chief medical officer^b^	Medicaid health plan		Multiple regions		5001-10,000	
**Health care providers or leaders**							
	Director of telehealth, specialty care provider	Safety net health care system		West		5001-10,000	
	Chief medical informatics officer, primary care provider	Safety net health care system		Midwest		5001-10,000	
	Clinic director, primary care provider	Safety net health care system		West		5001-10,000	
	Director of digital innovation, specialty care provider	Academic medical center		West		>10,000	
	Quality improvement lead, primary care provider	Academic medical center		West		>10,000	
	Director of biomedical informatics	Academic medical center		Northeast		1001-5000	

^a^The individual participated in the interview only.

^b^The individual participated in the survey only.

### Qualitative Interview Findings

#### Overview

Qualitative analysis findings of the in-depth interviews by CFIR domain, with a full set of exemplar quotes, are provided in Multimedia Appendix 2 (Tables S1-S4). There were some domains with alignment across stakeholder groups alongside those with significant misalignment of priorities and perceptions. Where misalignment was observed, it took the form of the perceptions and priorities of venture capitalists and digital health companies converging and those of payers and providers converging, with each pair expressing distinct viewpoints.

#### Outer Setting

All stakeholders described the need for changes to policy and the regulatory environment in order to drive widespread adoption of digital health tools. Current regulations were often cited as key barriers, for example,

The biggest challenge is really boring, but it’s regulatory...the billing and coding systems.

Digital health company

[Our decision making] relates to HIPAA [Health Insurance Portability and Accountability Act] as a very, very conservative approach about privacy and access.

Payer

Stakeholders also agreed that current financial mechanisms and processes are mismatched with goals and needs of patients and frontline providers. One digital health company participant summarized the overall misaligned priorities in the external environment:

If you’re a payer, you’re really thinking about optimizing your cost. If you’re a patient, you’re really thinking about optimizing your health which may cost more, right? If you’re a venture capitalist, you’re really thinking about return on your investment. So, in that sense, everybody, all of the objectives are slightly at odds and [not] quite right, but they’re not all aligned perfectly.

Furthermore, venture capitalists and digital health companies emphasized the need for immediate financial return and the simultaneous desire to disrupt the system in a fundamental way. One venture capitalist participant stated,

If this [product] isn’t something where you’re trying to maximize margins or drive sales, drive revenue, then it might make sense for you to think about other forms of capital.

Similarly, a participant from a digital health company stated,

There’s a lot of pressure to grow very fast, and it doesn’t take into account the mission of the organization, because of what the [financial] opportunity is.

In contrast, while payers and providers mentioned the need for external incentives to drive patient-facing digital health uptake, they commented in a more balanced way on the need for digital products that improve incrementally as well as those that are disruptive. One provider summarized this, saying,

There are some companies that are aligned with trying to sell into what our business model is and then there are others who are trying to reinvent health care in terms of how health care is delivered, how health care is paid for.

#### Inner Setting

Stakeholders agreed that frontline perspectives from patients and providers are valuable but not often obtained. In addition, most stakeholder groups pointed to the need to improve integration of patient-facing digital tools into provider workflows. For example, interviewees stated,

I think we all know that it is important to also make sure that you’re educating and getting feedback from the boots on the ground that are actually going to be using it, but...it’s really easy to just focus on getting the contract signed and getting the executive buy-in.

Venture capitalist

I think a lot of the digital health and IT [information technology] implementation...is more trickledown... At least in my experience, I don’t think that there has been much of an outreach to get provider opinion about these things before going in.

Health care provider

However, within this domain, the providers particularly emphasized lack of provider workflow integration as key barrier:

The reality is no one wants to open a second program to try to do something when they’re busy and they’re trying to do things...Maybe even more so for providers, for doctors, nurse practitioners and people like that who have hard-to-change behavior already.

Finally, payers and providers also offered concrete examples of bandwidth and staffing challenges to advancing patient adoption of digital tools. For example, interviewees stated,

We have bandwidth issues. We have things that we have to do because the state tells us, or some of our big providers...say it’s a priority for them. It’s being able to eke out enough space to work on something that might well be considered discretionary.

Payer

There may be negative bandwidth to do that kind of stuff...this small piece of integration to get the reports in our [EHR] system.

Health care provider

#### Intervention Characteristics

With respect to the characteristics of the digital health tools themselves, most interviewees mentioned the need for better functionality, particularly in terms of ease of use and seamless data sharing.

Number one, it has to be incredibly simple and intuitive.

Digital health company

[There is a lack of] a seamless end-to-end continuum [of data sharing/transmission] that can enable a person’s longitudinal health over time.

Payer

However, the largest differences between stakeholders within this domain were related to evidence about the uptake and effectiveness of the digital health tools. Payers and providers particularly emphasized the lack of sufficient evidence to show that large groups of patients would use available digital tools, as well as a lack of evidence demonstrating clinical benefits:

The [self-insured] employers say, “Almost no one’s using them [digital health tools],” and then the vendor [digital health company] is saying, “Well, yes, our engagement rates are 75%.” So I think there’s a big disconnect between what the vendors think is possible and what the reality is of the employees either finding or wanting to use these tools.

Payer

If we put this app there as a benefit, you got to feel reasonably confident it’s going to have...a reasonable likelihood to benefit. What’s the evidence for that?

Payer

#### Characteristics of Individuals

Finally, when considering both patient and provider characteristics, all groups mentioned their desire to focus meaningfully on patient engagement with digital health tools to make the largest impact in the digital health ecosystem. For example, many interviewees mentioned patient age (ie, older patients) and lower behavioral readiness as factors inhibiting use, suggesting that certain subgroups of patients need targeted approaches to achieve broad uptake. For example, interviewees stated,

These apps are still on the whole for techies, so it’s hard when you start getting into populations like the whole senior population.

Venture capitalist

[If] the [patients] that don’t want to engage at all, the solution obviously cannot reflect the need that they have, and that’s the gap that we’ve seen.

Payer

Most interviewees also suggested that patient uptake depends on provider engagement and recommendation of digital health tools. Despite this, they offered few examples or concrete strategies for how to work with providers to promote patient engagement. For example, interviewees stated:

If the patient knows that someone or that their doc is following along, they’re more likely to remain engaged.

Digital health company

I tend to avoid giving patient recommendations [for any app or digital platform], except for a few things I’ve specifically looked at where I trust the source.

Health care provider

Overall, venture capitalists and digital health companies described generic patient interest and ability to use digital health tools, while the providers in particular gave specific examples of patient factors that impede digital platform use, such as digital and health literacy and language accessibility. This contrast was exemplified in quotations:

I think making the individual who has the chronic condition the center of all of the efforts is key... [Consider] the barriers to people living in the most healthful way possible.

Digital health company

I think English speaking is a pretty big piece of that because most of the apps are not going to be available in necessarily multiple languages, certainly not beyond probably Chinese, Spanish—probably not even beyond Spanish.

Health care provider

#### Overarching Qualitative Themes

Synthesizing across CFIR domains revealed 3 themes—(1) Patient needs and preferences are secondary, (2) lack of shared definition of success blocks progress, and (3) each stakeholder group focuses on immediate but diverging priorities.

#### Patient Needs and Preferences Are Secondary

While all stakeholder groups believe it is important for the digital health sector to design for patients who are diverse in backgrounds, needs, and preferences, few put this into practice and consider how tools should be altered to accommodate varied patient characteristics. Venture capitalists and digital health companies focused more on outer setting (eg, reimbursement, regulation) and intervention characteristics (eg, app functionality) for mass market dissemination. While payers recognized barriers to patient use of digital health tools, they also lacked strategies to achieve meaningful patient engagement. Finally, while providers were best able to articulate the needs of individual patients (such as variation by age, socioeconomic status, and digital literacy), they had little bandwidth or ability to recommend digital tools to their patients or integrate digital tool use in practice.

#### Lack of Shared Definition of Success Blocks Progress

There is a lack of evaluation of digital health tools, with payers and providers stating that there are no clear patient-facing tools to recommend based on evidence. Underscoring this is a misalignment between stakeholder groups’ definitions of digital health platform effectiveness or success. Differing definitions of success can lead to different stakeholders focusing on different outcomes, which divides focus and therefore impedes progress. This was evident when digital health companies focused on high-level utilization data, such as the total number of downloads or users of their platform, while providers and payers focused on behavioral or outcome measures, including who was offered versus who adopted the technology and examination of adoption overall, as well as, by key patient groups (eg, uptake of tools only among younger, healthier populations who are already engaged in the healthcare system, also known as the “worried well”).

#### Each Stakeholder Group Focuses on Immediate but Diverging Priorities

There are large differences between stakeholder groups in how they view the most pressing needs within the patient-facing digital health space. For providers, it was clear that lack of integration into electronic health record systems and workflows are a huge challenge to adoption. This inner setting challenge, in turn, leads providers to be less willing to recommend tools to their patients and want to communicate about use of digital tools in the context of their existing care, missing an opportunity to drive patient engagement. Digital health companies and venture capitalists focused most on the outer setting challenges such as regulatory compliance including billing and coding systems, privacy, and incentives that prevent uptake of digital health tools.

### Quantitative Survey Findings

**Figure 2:**
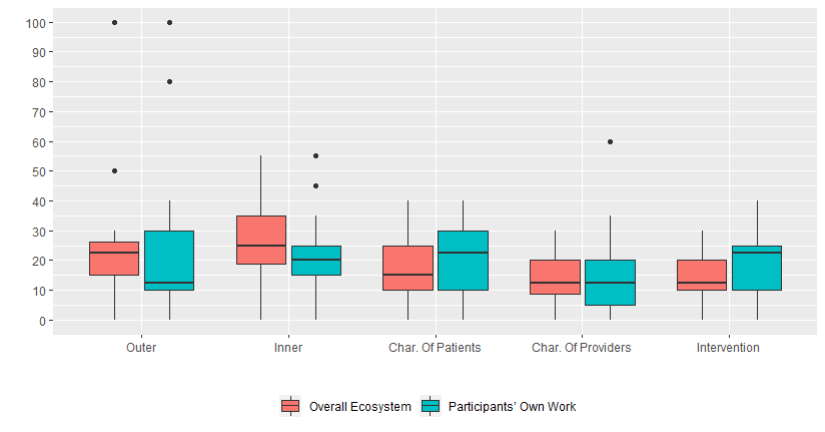
Responses by Consolidated Framework for Implementation Research domain. Char: Characteristics.

When asked to consider the CFIR domains in a structured, comparative way via survey, all CFIR domains were perceived as important drivers of the patient-facing digital health ecosystem. Point allocation among participants was highest for the outer setting and inner setting in the overall ecosystem. Scores differed somewhat when we asked for allocations in the participants’ own work*,* with slightly higher median scores in the inner setting, characteristics of patients, and intervention domains (Figure 2).

**Figure 3:**
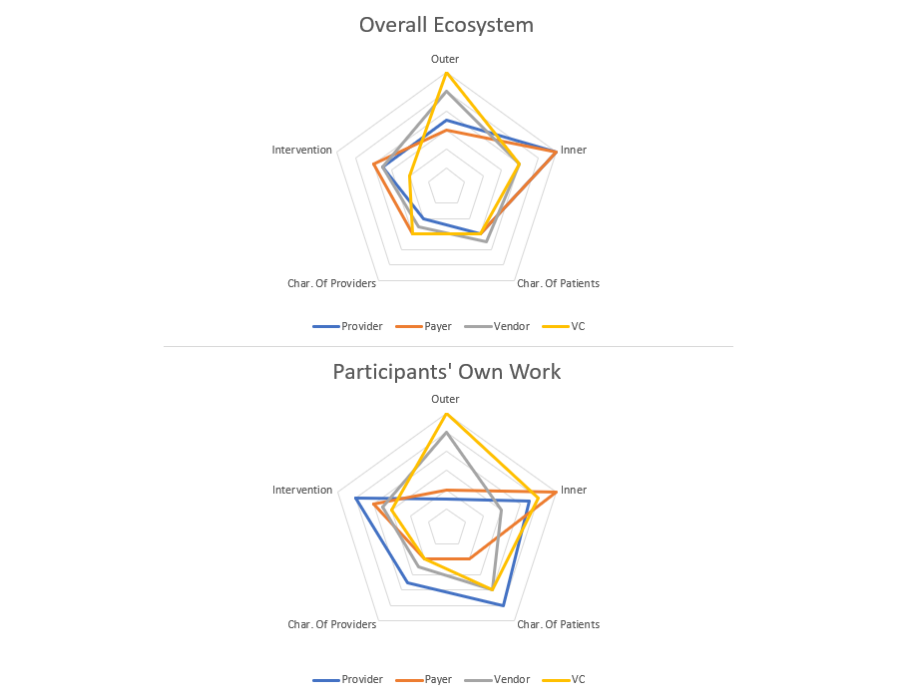
Radar chart showing responses by stakeholder group. Char: Characteristics; VC: venture capitalist.

We found evidence of stakeholder misalignment that was consistent with findings from the interviews. From radar charts (Figure 3), we observed that digital health companies and venture capitalists were aligned in their assessment, and emphasized outer setting as posing a challenge to the patient-facing digital health ecosystem and as the domain with the largest effect on their individual work. Providers and payers were aligned in their assessment of the importance of the inner setting in the overall patient-facing digital health ecosystem, yet providers were most likely to focus on patient characteristics in their own work. Venture capitalists had significantly lower scores for the intervention domain within the digital health ecosystem than payers (*P*=.02) and providers (*P*=.03). Providers had significantly lower scores for the outer setting domain within the individual work question than venture capitalists (*P*=.049) and digital health companies (*P*=.02).

## Discussion

### Principal Findings

While private-sector digital health solutions that are implemented by health plans and health care delivery systems are widely touted as a key driver of health system transformation [25,26], our study revealed overlap in some conceptual domains along with clear gaps between these sectors. All stakeholder groups (venture capitalists, digital health companies, payers, and providers) identified frontline provider, staff, and patient engagement as drivers of widespread uptake of patient-facing digital tools. Yet our analyses of key informant interviews and survey results revealed misalignment between the focuses of these groups that are likely impeding the ability to achieve that uptake. Specifically, venture capitalists and digital health companies often focused on issues in the outer setting (eg, regulation). In contrast, provider and payer interviews largely focused on issues in the inner setting (eg, workflows). These overall emphasis areas were maintained in our follow-up surveys with stakeholders as they ranked the priorities of various implementation domains in their daily work. Taken together, as we synthesized findings into broad themes, we found that patient characteristics (eg, needs and preferences related to digital health tool use) were often addressed secondarily in the digital health ecosystem.

Our work sheds light on the specific nature of the disconnect within this ecosystem, on which other studies have suggested similar concepts. For example, recent expert synthesis to advance the digital health landscape has also called for stronger evidence of clinical improvements from digital health apps and more careful consideration of workflow integration to drive widespread implementation [27]. Similarly, consensus reports from stakeholder convenings, such as reports from influential organizations like the World Health Organization [17], also support the need for bringing diverse stakeholders together to set and carry out shared priorities. Furthermore, a large body of patient-facing research on barriers to digital health tool use highlights digital literacy barriers and the desire to use technology that augments their existing provider and staff relationships [6], which is consistent with our findings. Our study makes a unique contribution by collecting and analyzing primary data from a diverse set of stakeholders and focuses on multiple rather than a single stakeholder group [28-31]; therefore, our analyses add detail to the problems discussed in the field, informed by an implementation framework, that could advance our next steps in a multilevel fashion.

### Policy and Practice Implications

Moving forward, the diverging priorities across stakeholder groups will make it difficult to implement, spread, and sustainably reimburse patient-facing digital health tools in real-world health care settings. Our work suggests a role for increased communication among these stakeholders, as each stakeholder group lacked detailed understanding of other groups’ immediate priorities. If federal and state policy could remove barriers in the outer setting, then venture capitalists and companies may be better able to anticipate regulatory environments that protect patients yet move and adapt at the appropriate pace to support new innovation. Recent evidence suggests major players such as the Federal Drug Administration and the Centers for Medicare and Medicaid Services are fostering change in this space [32]. These efforts by key policy stakeholders have promoted the growth of digital tools, primarily by increasing patients’ ability to access their health data in electronic formats that can be connected to smartphones and other platforms. In parallel, these entities have also pursued more oversight over tools—in particular those that act as medical devices to help ensure safety and efficacy. In turn, these efforts could promote consumer confidence and broader adoption. However, the outer setting issues identified related to reimbursement and regulation were broad—spanning from the need for broader value-based payment to privacy or security challenges to regulation of novel technologies (eg, artificial intelligence). While federal policymakers are tackling each of these areas, these efforts are not guided by a singular focus on advancing digital health and so the results are likely to be uneven.

Moreover, these outer setting changes will likely not succeed without simultaneous focus on inner setting barriers, such as integration into workflows and increased focus on provider and patient needs [33]. Even in light of the COVID-19 pandemic, during which many patient-facing digital tools are rapidly being tested to support patients remotely, a dearth of workflows to support their use—coupled with lack of evidence generation about health improvements and exacerbation of health disparities—could continue to suppress wide scale adoption in the long term [34,35]. In particular, business models for digital health companies will diverge from frontline health care system needs until these stakeholders can tie return on investment to meaningful and immediate priorities for patient care. Therefore, direct work across stakeholders to create a shared agenda for working more closely together will be critical to create alignment. All stakeholders must be willing to jointly decide (from pilots to large scale implementation) on (1) reimbursement/business models that incentivize diverse patient uptake of tools; (2) standard workflows and processes for piloting tools within real-world settings; (3) evaluation metrics that address uptake, engagement, and clinical effectiveness; and (4) policy and regulatory oversight that maximizes speed while maintaining quality and safety of digital approaches.

### Limitations

There are several limitations of this study that are important to note. First, the sample size was modest and overrepresents stakeholders in the western United States and academic medical centers in urban and suburban areas (likely driven by both the sampling strategy and the geographic clustering of venture capitalist firms and digital health companies). Furthermore, we did not explicitly sample digital health researchers or patients in this study, given our decision to focus on privately developed digital health tools and stakeholder groups that were less represented in previously published literature. Future work with a broader sampling approach across all stakeholder groups is needed. However, our mixed methods approach likely increased the potential generalizability of our findings. In addition, we focused our analysis on top-level CFIR domains rather than on CFIR subdomains, given the large misalignment that was evident at the highest domain levels in our qualitative coding. Future work is needed to flesh out nuanced barriers and facilitators at the subdomain level.

### Conclusions

Despite the presence of some overlapping perspectives across stakeholder group priorities, the gaps and misalignment between digital health companies and their funders, on one hand, and providers and payers, on the other, deserves direct attention in striving for digital transformation. Closer, longitudinal collaboration among stakeholders and team-based approaches may address this fundamental challenge—especially to ensure that digital solutions are matched to the needs of the diverse US population [36].

## Appendix

Multimedia Appendix 1 Interview guide.

Multimedia Appendix 2 Example quotations (by CFIR domain).

## Abbreviations

CFIR: Consolidated Framework for Implementation Research
