# Supplementary material for: Alignment of Key Stakeholders’ Priorities for Patient-Facing Tools in Digital Health: Mixed Methods Study
Source: J Med Internet Res. 2021 Aug 26;23(8):e24890. doi: 10.2196/24890 (PMC8430871; doi:10.2196/24890)
Supplement: Multimedia Appendix 1 [file jmir_v23i8e24890_app1.docx]

Appendix 1. Interview Guide

**Interviewee and Organization Information**

1. Please describe your role and how long you have worked in it
2. Please tell us about your organization.
   1. Years in operation
   2. Mission/ goal
   3. INVESTORS: size of digital health portfolio and focus of digital health investments (e.g. maturity of companies they invest in, social impact focus)

As we mentioned, we are trying to characterize alignment and misalignment around digital health among different stakeholder groups (e.g. entrepreneurs, developers, investors, providers, health systems leaders, payers, patients):

**Questions**

1. What do you think is the biggest misalignment in digital health, broadly? In other words, what are the gaps between what is being built/ funded vs. what patients/ providers need?
   1. PROBES:
      1. Is this affected by varying definitions of digital health? What does the term mean to you, and how might it vary among different stakeholder groups?
      2. How might this depend on different standards for evidence strength and quality? (INTERVENTION CHARACTERISTICS)
      3. Has alignment changed over time? What has changed? Why do you think this has happened? (OUTER AND INNER SETTING)
      4. How might your position within the digital health ecosystem influence your perception of alignment/ misalignment? E.g. provider often seeing patients needs not being met by digital health products (CHARACTERISTICS OF INDIVIDUALS)
      5. How might the varying processes of implementing digital health affect this alignment/ misalignment? E.g. individual implemented at health system level (PROCESS)
      6. (If not already addressed) What is the biggest alignment/misalignment for patients?
         1. How might this vary by patient type, demographics, setting (e.g. Medicaid)
2. What promotes use of a digital health product? How do you assess user needs? How do you measure use/ usability?
   1. PROBES:
      1. How might this vary among products that are patient vs. system facing? Or by product features (e.g. passive data collection, integration) (INTERVENTION CHARACTERISTICS)
      2. Are there any external factors that cause you to prioritize some user needs over others? (OUTER SETTING)
      3. What about by the implementation setting itself (e.g. culture for change, resources)? (INNER SETTING)
      4. How might they vary by user type (e.g. demographics, motivation, knowledge and beliefs about the intervention)? (CHARACTERISTICS OF INDIVIDUALS)
      5. How might this vary by the way the product is implemented, and who is involved in that implementation? (e.g. dedicated onboarding team, implementation leaders, champions) (PROCESS)
3. What causes digital health to ‘stick’ in your experience (e.g. move from pilot to scalable, sustainable product embedded in workflow)?
   1. PROBES:
      1. How much has to do with Integrations/ interoperability? (INTERVENTION CHARACTERISTICS)
      2. What about to policies/ incentives? Or relationships with corporations (e.g. Epic)? (OUTER SETTING)
      3. How might scaling and sustainability be affected by existing workflows? How might it vary by implementation site (e.g. a Medicaid setting)? (INNER SETTING)
         1. What do you see as the challenges/ untapped opportunities in under-resourced settings?
      4. How is this affected by individuals’ attitudes towards change/ technical readiness/ sense of self-efficacy? (CHARACTERISTICS OF INDIVIDUALS)
      5. And how might be affected by who is involved (e.g. CMO, what your colleagues use, etc.) (PROCESS)
4. Finally, what do you think about when deciding to build/ purchase/ use/ invest in a digital health product?
   1. PROBES:
      1. Level of evidence (effectiveness/efficacy as well as adoption/uptake) (INTERVENTION CHARACTERISTICS)
      2. Interoperability (INTERVENTION CHARACTERISTICS)
      3. Cost (INTERVENTION CHARACTERISTICS)
      4. Policies/ incentives (OUTER SETTING)
      5. Alignment with existing workflows (INNER SETTING)
      6. How might this be different than what your colleagues think (those within your stakeholder group)? Do you think that other stakeholders in the digital health space (e.g. payers, investors, providers, patients, digital health developers) use similar criteria to evaluate promising/successful digital health platforms? (CHARACTERISTICS OF INDIVIDUALS)
      7. Whether or not there are champions, dedicated resources to support implementation? (PROCESS)
