# Supplementary material for: Alignment of Key Stakeholders’ Priorities for Patient-Facing Tools in Digital Health: Mixed Methods Study
Source: J Med Internet Res. 2021 Aug 26;23(8):e24890. doi: 10.2196/24890 (PMC8430871; doi:10.2196/24890)
Supplement: Multimedia Appendix 2 [file jmir_v23i8e24890_app2.docx]

Appendix 2. Exemplar Quotes by CFIR Domain

| **Table S1. Exemplar Quotes by CFIR Domain – Outer Setting** | **VC participant quote** | **Digital health company participant quote** | **Payer participant quote** | **Provider participant quote** |
| --- | --- | --- | --- | --- |
| Misalignment of business models | There is no formula, although it does have to answer those two that I said before; with the plausible future that returns the fund, and the second is, “What’s the impact over 30-50 years?” | If you’re a payer, you’re really thinking about optimizing your cost. If you’re a patient, you’re really thinking about optimizing your health which may cost more, right? If you’re a venture capitalist, you’re really thinking about return on your investment. So, in that sense, everybody, all of the objectives are slightly at odds and [not] quite right, but they’re not all aligned perfectly. | The business of health plan is they get money. They create a network. They receive claims and they make payments and then they do utilization management, adjudication, sometimes case management, and some pharmacy adjudication. Oftentimes in a health plan, what a thing like a [digital health tool] is adjudicated, I guess, is how did it help you do your core business? … The things related to patient interactions were little more secondary. | There are some companies that are aligned with trying to sell into what our business model is and then there are others who are trying to reinvent healthcare in terms of how healthcare is delivered, how healthcare is paid for. That’s one place where there’s potential misalignment is there’s just a lot of companies who are essentially trying to put us out of business. |
|  | I mean it’s an astonishing legacy payment system that is – it creates the things that are profitable but are not the right things to give to the patient. | One is if you’re super successful, then there’s a lot of pressure to grow very fast, and it doesn’t take into account the mission of the organization, because of what the opportunity is, right, and funding and capitalism is what it is. |  |  |
|  | If it’s going to be capital in terms of business, you’re going to be required to continue to raise funds and more and more often, as you grow as a company and as you evolve…. If this [product] isn’t something where you’re trying to maximize margins or drive sales, drive revenue, then it might make sense for you to think about other forms of capital. |  |  |  |
| Challenges with regulation | So, why don’t we live in a world where once something is well known and true but it doesn’t get distributed and done better?...I think it’s a level of protectionism. There’s probably a level of conservatism [in regulation/policy]…In general, you get in this sporadic system that is a big gridlock with all these overlapping interests and oligopolistic interests. | The biggest challenge is really boring, but it’s regulatory. At the end of the day, it’s like until the billing and coding systems [work.] | It all relates to HIPAA as a very, very conservative approach about privacy and access. They’ll [state Medicaid programs] hold conferences about how we want to do stuff, but when it comes down to actually approving our operations, it comes to a very strict approach to the regulation and interpretation. | Some of the devices Medicare won’t pay for. So, if you’re a Medicare patient, I’ll have to pick a particular device for you, and then if I pick that device for you, you have to use a particular software. |
|  | I think realistically, you so often see in healthcare and other industries that regulation ends up lagging behind, both workflow and technology innovation because they’re just not real time workflows. | I think some of them [payers] understand the potential, but I don’t know, I think they’re not all moving into the game as rapidly as they could or should and perhaps that’s because it’s not clear if they should be reimbursing, if there are claims that can be filed for the use of the tool. | When we, at the health plan, are putting this out to our members, then it’s required of us that we know that our vendor has safeguards in place and policies and practices that are restrictive and robust in ways in which we internally have to handle member PHI [protected health information]. | Getting those [federal] approvals, getting the algorithms, how the data [work] - if you’re going to invoke artificial intelligence, those algorithms have to be not only validated but validated on a medical and safety level. That’s a huge endeavor. |

| **Table S2. Exemplar Quotes by CFIR Domain – Inner Setting** | **VC participant quote** | **Digital health company participant quote** | **Payer participant quote** | **Provider participant quote** |
| --- | --- | --- | --- | --- |
| Attention on leadership buy-in/rollout rather than frontline engagement | What I will say though is that often from a small company perspective, you are told by everyone around you that you need to get to the C-suite…I think we all know that it is important to also make sure that you’re educating and getting feedback from the boots on the ground that are actually going to be using it, but…it’s really easy to just focus on getting the contract signed and getting the executive buy-in. | If there isn’t a really strong clinical champion then these tools usually wither on the vine during the process of a pilot. | [We don’t find digital health tools] through an efficient: “These are the best things. Only the best things are being presented in front of you.” Unfortunately. Sometimes it’s…a referral from this person….Sometimes it’s like, “Oh, my God. We got this huge problem,” and then we start looking. | I think a lot of the digital health and IT [information technology] implementation…is more trickledown….At least in my experience, I don’t think that there has been much of an outreach to get provider opinion about these things before going in. |
|  |  |  |  | In a large organization like ours, there’s sometimes a disconnect between the system-wide strategic initiative and what we need to do locally. |
| Challenges with workflows | I would say that’s the other blind spot for a lot of entrepreneurs: how well that fits in into the clinical workflow. Because…they’re like, “Of course, the hospital will buy it,” but the reality is that that’s not true at all. It has to be tried. It has to be adopted. It has to be certified, and it has to inter-operate with everything else. | Ultimately, when you’re asked about key aspects of feasibility, one of the biggest potential issues around feasibility is just how easy it is to get up and running. From let’s say the time a contract is signed, now there’s an entire process just trying to get a contract signed with a hospital, which is a very lengthy process, but if you start from the time a contract is signed, you want to minimize the amount of time a customer has to wait until you go live with your first patient and there are a lot of steps involved to getting something launched to go live. | In our organization, we’ve got to make the internal case to devote resources, whether it’s financial or staff, to move forward [with a digital product]. We have bandwidth issues. We have things that we have to do because the state tells us, or some of our big providers…say it’s a priority for them. It’s being able to eke out enough space to work on something that might well be considered discretionary. | For example, if I came up with an idea, “This is a great app. The one missing link to make this more functional…would be this small piece of integration to get the reports in our [EHR] system.”…There may be *negative* bandwidth to do that kind of stuff. |
|  | I think a lot of what we need to talk about is more broadly how do we think about the integration of technology and human care providers. |  |  | I think the integration piece is really important because I think the reality is no one wants to open a second program to try to do something when they’re busy and they’re trying to do things, and definitely, nobody wants to open up a scanned document and have to read blood pressure values off of that….Maybe even more so for providers, for doctors, nurse practitioners and people like that who have hard-to-change behavior already. |

| **Table S3. Exemplar Quotes by CFIR Domain – Intervention Characteristics** | **VC participant quote** | **Digital health company participant quote** | **Payer participant quote** | **Provider participant quote** |
| --- | --- | --- | --- | --- |
| Ease of use | It’s also usability, it’s design, and it’s awareness among the public that those offerings exist and are accessible, and that they get very high ratings from users. | Number one, it has to be incredibly simple and intuitive. Think about the iPhone design. You can build lots and lots of complexity into tools, but if it’s not very simple, intuitive, and actually delightful to use, people, they’re not going to sustain usage. | They’ll [digital health tools] specialize and address a very specific need but they’re not moving into a seamless end-to-end continuum that can enable a person’s longitudinal health over time. | Four different apps that all don’t talk to each other and are each sending separate reminders to “Take your meds, or “Enter your mood.” Those data should be entered once and then they spread everywhere. I think it has to move in that direction. |
|  |  |  |  | On the patient-facing side, that has to be low literacy and interfaces easily with the EMR [electronic medical record]. |
| Data integration |  | I think that there’s one problem that there’s a lot of standalone, analytical tools that don’t necessarily work broadly across every single device. Then, so one problem is aggregating data from multiple different devices and making meaning of it, and for different spaces, and different areas of someone’s health. | None of the tools that the employers are using integrate with anything else and that’s something that they were not happy with. I mean, I think in general, the employers are not happy with integration. | Because all these applications do is give information. That’s really what the level we are at now. That application is not going to allow them to converse with me or one of our team. There’s still going to be a phone call that’s initiated. |
| Evidence of digital tool’s benefit | I just found that most [digital health tools] to be to be widely available, highly variable in their quality, but with very, very low gauge rate…I mean that’s part of my biggest disappointment or surprise. | So, like you can train like patients to use digital products and then they learn, but then it falls apart, because it’s really not integrated into their life. It’s important for the person who built it and the provider, but not to the consumer. | The [self-insured] employers say, “Almost no one’s using them,” and then the vendor is saying, “Well, yes, our engagement rates are 75%.” So I think there’s a big disconnect between what the vendors think is possible and what the reality is of the employees either finding or wanting to use these tools. | “I mean, one, what’s the real value of the tool? Again, as I would say, in my space, I haven’t really seen a lot of tools where there’s proven value. Again, there’s a lot of vendors out there that give a good pitch or give a good story, but it’s really hard to find data.” |
|  | “But having a technology that you believe should exist and having a technology that actually works in a real world setting is totally different.” | Can you optimize someone’s lifestyle if you had 100% data?” So, let’s say we say, “Yes, we can, because we have these machine learning methods that change over time for each individual.” | If we put this app there as a benefit, you got to feel reasonably confident it’s going to have – if you do it, you have a reasonable likelihood to benefit. What’s the evidence for that? | I think the challenge with that is that, many times, we’re solving problems that people don’t really have. We’re telling them, “Here’s a product that’ll make your life better,” but we’re not really sure if it actually will. |
|  |  |  | They’ll [digital health companies] usually have some claim about how much they can reduce this or that or improve this or that, but we need to see some real evidence that this is the case…We have responsibilities in health plans and not put something else, some app there that’s pretty untested or experimental. |  |

| **Table S4. Exemplar Quotes by CFIR Domain – Patient and Provider Characteristics** | **VC participant quote** | **Digital health company participant quote** | **Payer participant quote** | **Provider participant quote** |
| --- | --- | --- | --- | --- |
| Patient motivation/interest and skills | So, when it comes to the patients themselves, I think people have drastically over-estimated the smartphone per se. People forget lessons we already know about the way people do and don’t want to interact with them. | They [patients] might not have access to a physician or to a diabetes educator, but they have access to a cellphone, even most often a smartphone. | But traditionally speaking, when we have the services available, it’s in our network and they can they [patients] can avail themselves of it. | I think all of us, myself included, have been overly optimistic. Optimistic isn’t even the right word. We forget how everyone has busy lives and that we see people as “patients,” but then they go back home. |
|  | These apps are still on the whole for techies, so it’s hard when you start getting into populations like the whole senior population. | The easy answer is someone who’s not using it is someone who’s not ready to make a change. | [If] the [patients] that don’t want to engage at all, the solution obviously cannot reflect the need that they have, and that’s the gap that we’ve seen. | It’s just health literacy, digital literacy, yes. Most of our patients have low literacy and of those, some are completely illiterate and then yes, the digital literacy on top of that. |
|  |  | I think making the individual who has the chronic condition the center of all of the efforts is key. Some examples, if you just think about levels of understanding of what are the barriers to people living in the most healthful way possible. | What I think we’re finding early on is the people that are preinclined towards naturally healthy behaviors would double down and use the incentives, but the ones that aren’t necessarily that engaged on the health topic continues to be a challenge regardless of the incentives. | I think English speaking is a pretty big piece of that because most of the apps are not going to be available in necessarily multiple languages, certainly not beyond probably Chinese, Spanish – probably not even beyond Spanish. |
| Need for provider awareness/engagement to successfully reach patients | Providers, if they see something that can make a big difference…and has a potential in being seamlessly adopted, they will go for it. But if it requires any additional training or change to the workflow or interoperability headaches, they will stall. | If the patient knows that someone or that their doc is following along, they’re more likely to remain engaged. | What our data shows is that the best way to engage the consumers is through their primary care doctors or through the delivery network in general. | I tend to avoid giving patient recommendations [for any app or digital platform], except for a few things I’ve specifically looked at where I trust the source. |
|  | I haven’t thought of an instance [where a provider would say], “Oh my gosh, if I could use that tool, would that make the care a lot better, relative to what I’m doing? Already, patients can email and text me, or I can talk to them.” | Awareness gap is on the professional side. Frankly, there are so many digital health tools out there that there’s no way that a clinician is aware of all of them or even aware of which ones in fact should be [used]. |  | There’s bias in who we [providers] bring it up with, right? So the providers are bringing it [digital health tools] up with people who you think are going to be receptive to it. |
